# Supplementary material for: Axenic Culture of Caenorhabditis elegans Alters Lysosomal/Proteasomal Balance and Increases Neuropeptide Expression
Source: Int J Mol Sci. 2022 Sep 29;23(19):11517. doi: 10.3390/ijms231911517 (PMC9570027; doi:10.3390/ijms231911517)
Supplement: Supplementary file 1 [file ijms-23-11517-s001.zip › ijms-1919799-supplementary.pdf]

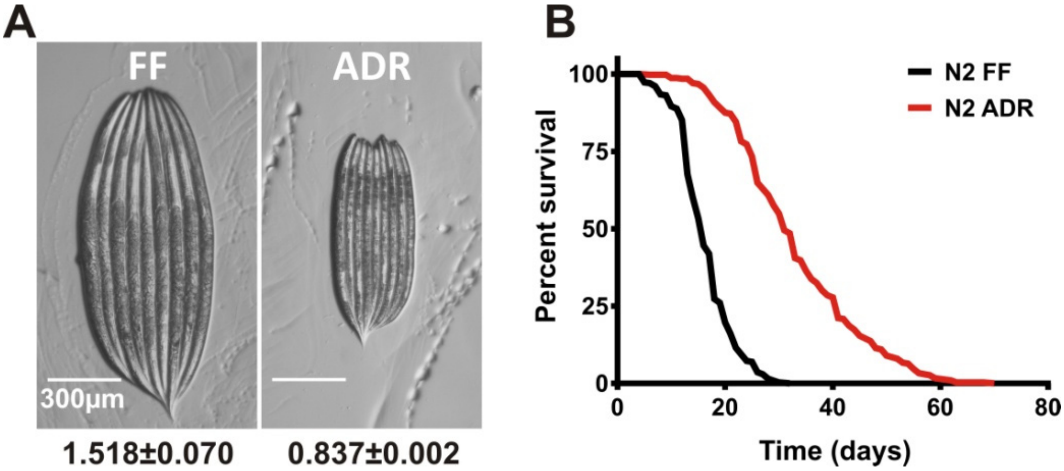

Figure S1. Habitus and survival of FF and ADR adult worms. (A) Nomarski DIC microscopy of FF and ADR worms; the average body volume in nL is indicated below the micrographs (mean± s.e.m). Volume was estimated using the cylinder formula and the measurements of worm length (1152.97±5.41 and 933±6.63 µm for FF and ADR worms respectively) and width (40.93±1.27 and 33.8±0.10 µm for FF and ADR respectively). (B) Lifespan analysis of FF and ADR worms with mean lifespan, 16.45±0.27 and 33.23±0.65 (mean± s.e.m), respectively. FF = fully fed, ADR = axenic dietary restriction.

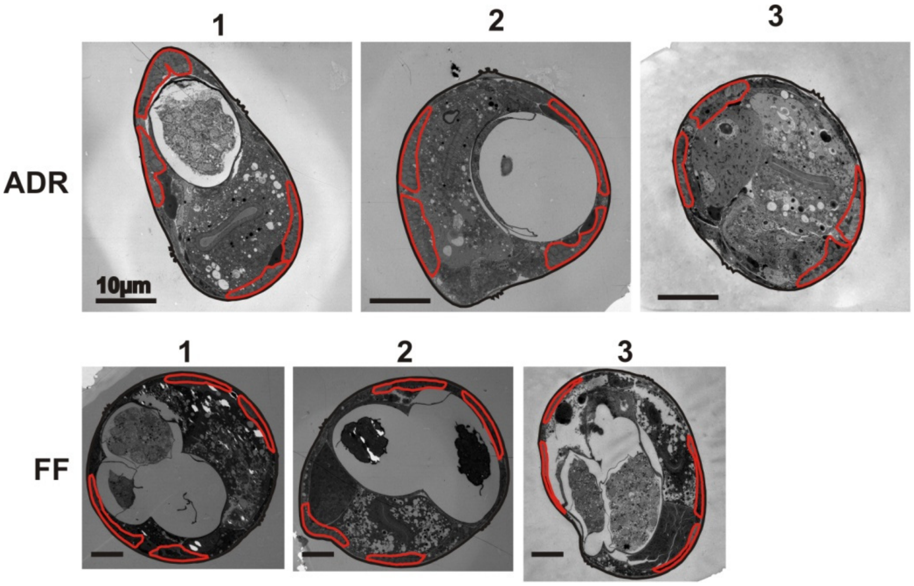

Figure S2. ADR worms have elevated muscle mass compared to fully-fed controls. Muscle cells were marked as red area of the total cross section of the worm mid-body (TEM images). Muscle surface area (in red) was determined using Image J software and was normalized to total section area.

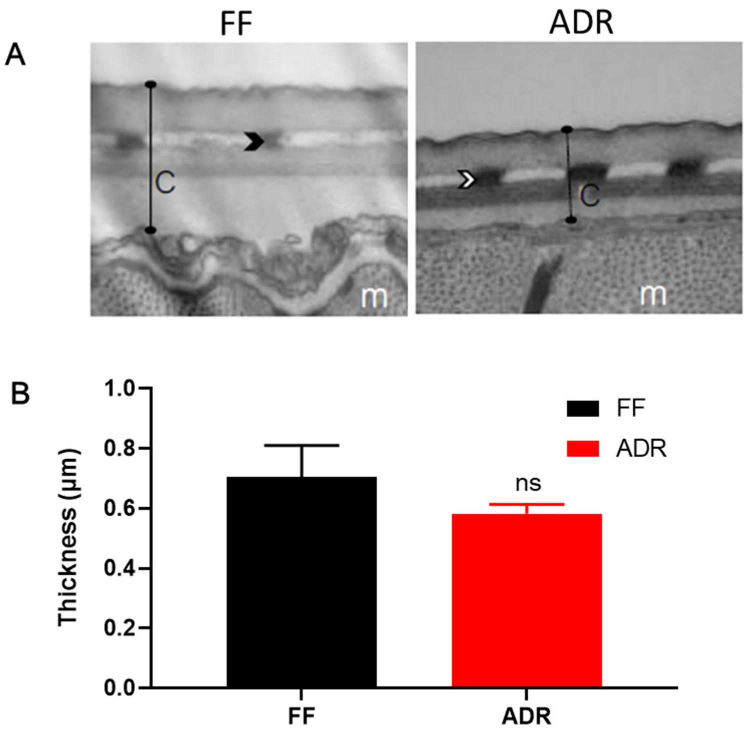

Figure S3. (A) TEM images of cuticles of worms grown in FF (fully fed) and ADR (axenic dietary restriction) conditions. (B) Cuticle thickness in FF (fully fed) and ADR (axenic dietary restriction) worms. P=0.1077. ns: not significant

Supplemental Table S1. Gene ontology information from DAVID bioinformatic resource of DEGs. Reported *P* value is the modified Fisher Exact *P* value or EASE score.

**Representative term (GO, INTERPRO, …)**	Enrichment score	*P* value
**Upregulated in axenically cultured worms**		
IPR006210:EGF-like	7.73	1.56E-09
GO:0044421~extracellular region part	6.39	2.26E-07
GO:0031032~actomyosin structure organization	5.87	6.28E-08
IPR018159:Spectrin/alpha-actinin	5.48	1.14E-06
IPR013091:EGF calcium-binding	4.94	1.25E-06
IPR003599:Immunoglobulin subtype	4.59	7.41E-08
GO:0043292~contractile fiber	4.01	4.92E-05
IPR001781:Zinc finger, LIM-type	3.03	7.03E-04
GO:0030182~neuron differentiation	2.91	3.26E-05
IPR004009:Myosin, N-terminal, SH3-like	2.85	6.43E-04
laminin egf-like domain	2.84	1.45E-04
GO:0019098~reproductive behavior	2.80	5.61E-04
IPR002048:Calcium-binding EF-hand	2.76	3.02E-04
GO:0031034~myosin filament assembly	2.73	1.86E-03
GO:0008340~determination of adult life span	2.66	2.20E-03
GO:0006928~cell motion	2.62	1.70E-03
GO:0018988~molting cycle, protein-based cuticle	2.52	2.94E-03

GO:0009791~post-embryonic development	2.28	3.97E-03
IPR001791:Laminin G	2.24	4.18E-03
IPR015650:Heavy chain of Myosin	2.09	1.16E-03
IPR019748:FERM central domain	2.04	1.54E-03
IPR009007:Peptidase aspartic, catalytic	2.04	1.54E-03
domain:Immunoglobulin	1.98	1.24E-02
domain:LDL-receptor class A 1	1.95	1.13E-02
IPR003961:Fibronectin, type III	1.82	6.62E-03
IPR004827:Basic-leucine zipper (bZIP) transcription factor	1.82	3.27E-03
GO:0008233~peptidase activity	1.67	1.24E-02
IPR008160:Collagen triple helix repeat	1.63	9.96E-03
GO:0009374~biotin binding	1.62	3.75E-02
IPR017940:ABC transporter integral membrane type 1	1.60	2.12E-03
GO:0034622~cellular macromolecular complex assembly	1.31	2.01E-02
IPR011001:Saposin-like	1.31	3.63E-02
GO:0040008~regulation of growth	1.21	3.48E-02
IPR001192:Phospholipase C, phosphoinositol-specific, C-terminal (PLC)	1.18	3.29E-02
GO:0005874~microtubule	1.17	4.51E-02
GO:0043492~ATPase activity, coupled to movement of substances	1.16	1.33E-02
domain:EF-hand 1	1.12	3.89E-02
GO:0004857~enzyme inhibitor activity	1.08	4.53E-02
binding site:NAD	1.06	4.37E-02
**Downregulated in axenically cultured worms**		
GO:0005839~proteasome core complex	10.04	2.55E-12
GO:0000502~proteasome complex	9.32	3.33E-12
GO:0022402~cell cycle process	8.99	4.54E-10
GO:0000279~M phase	7.38	4.74E-09
GO:0007276~gamete generation	6.49	1.80E-07
GO:0030163~protein catabolic process	6.47	1.90E-09
GO:0009791~post-embryonic development	4.16	5.20E-05
IPR003959:ATPase, AAA-type, core	3.78	4.56E-06
GO:0040010~positive regulation of growth rate	3.73	1.21E-04
GO:0040021~hermaphrodite germ-line sex determination	3.71	7.14E-05
IPR006649:Like-Sm ribonucleoprotein, eukaryotic and archaea-type, core	3.52	2.18E-04
GO:0007548~sex differentiation	3.27	1.65E-04
IPR006671:Cyclin, N-terminal	3.22	2.18E-04
IPR016050:Proteasome, beta-type subunit, conserved site	2.92	3.96E-04
GO:0006281~DNA repair	2.81	7.57E-04
IPR016897:E3 ubiquitin ligase, SCF complex	2.65	2.93E-04
GO:0019787~small conjugating protein ligase activity	2.51	1.92E-03
IPR001680:WD40 repeat	2.31	9.73E-04
IPR015880:Zinc finger, C2H2-like	2.17	4.18E-03
GO:0060429~epithelium development	2.14	6.90E-03
GO:0000280~nuclear division	2.02	5.41E-03

IPR012677:Nucleotide-binding, alpha-beta plait	1.82	1.02E-02
GO:0048581~negative regulation of post-embryonic development	1.82	1.00E-02
GO:0032993~protein-DNA complex	1.81	8.36E-04
IPR014400:Cyclin, A/B/D/E	1.66	1.32E-03
GO:0007127~meiosis I	1.40	6.09E-02
IPR017937:Thioredoxin	1.32	7.55E-03
GO:0008406~gonad development	1.20	5.56E-02
GO:0008629~induction of apoptosis by intracellular signals	1.01	3.40E-02

Supplemental Table S2. Shortlist of the genes tested in lifespan assays.

Gene names	Gene ontology	Fold change	Mean lifespan (days)	*P*-value
**L4440**			**40.77±1.43**	
**Large fold change**				
col-94	structural constituent of cuticle	38.91	44.68±1.59	*P*=0.0141
tbb-6	GTPase activity; microtubule; microtubule-based process; protein polymerization; structural constituent of cytoskeleton	19.77	42.76±1.02	*P*=0.8521
T11B7.2		14.12	45.59±2.08	*P*=0.0895
F15D4.5	nucleic acid binding; zinc ion binding	12.88	34.3±2.68	*P*=0.0368
**Lysosomal genes**				
hch-1	metalloendopeptidase activity	27.66	49.7±1.55	*P*=0.0001
asp-5	aspartic peptidase	4.57	39.75±1.37	*P*=0.9613
asp-8	aspartic peptidase	6.56	37.42±1.33	*P*=0.0355
asah-1	acylsphingosine amidohydrolase	9.22	42.52±1.64	*P*=0.5556
pho-8	phosphatase	19.18	50.96±3.74	*P*=0.0119
**Amino acid metabolism**				
got-1.2	an ortholog of human glutamic-oxaloacetic transaminase 1	3.97	47.45±2.44	*P*=0.0316
gln-1	an ortholog of human glutamate-ammonia ligase	3.02	44.45±1.46	*P*=0.2156
F46H5.3	arginine kinase activity; ATP binding	2.99	42.92±1.35	*P*=0.3550
**Transcription factors and cofactors**				
nhr-62	nuclear hormone receptor	9.46	40.65±1.26	*P*=0.7906
nhr-132	nuclear hormone receptor	6.73	34.56±1.70	*P*=0.0013
ceh-37	homobox transcription factor	5.89	38.08±1.45	*P*=0.2656
nhr-90	nuclear hormone receptor	5.77	43.45±1.21	*P*=0.3845
nhr-6	nuclear hormone receptor	5.1	44.67±2.52	*P*=0.1257
lim-7	transcription factor	4.88	47.29±2.11	*P*=0.0139
nhr-45	nuclear hormone receptor	4.55	43.09±1.04	*P*=0.1889

nhr-120	nuclear hormone receptor	4.52	40.53±1.41	*P*=0.9712
nhr-84	nuclear hormone receptor	4.29	41.24±1.18	*P*=0.9612
xbp-1	ER stress transcription factor	2.4	34.70±1.80	*P*=0.0129
nhr-97	nuclear hormone receptor	2.17	40.35±2.22	*P*=0.8427

* Above RNAi experiment using the *rrf-3* strain. *P*-value compared to L4440.
